# Math Anxiety and Working Memory Updating: Difficulties in Retrieving Numerical Information From Working Memory

**DOI:** 10.3389/fpsyg.2020.00669

**Published:** 2020-04-09

**Authors:** Santiago Pelegrina, M. José Justicia-Galiano, M. Eva Martín-Puga, Rocío Linares

**Affiliations:** Department of Psychology, Faculty of Humanities and Educational Sciences, University of Jaén, Jaén, Spain

**Keywords:** math anxiety, working memory updating, working memory, memory retrieval, anxiety, updating components

## Abstract

This study aimed to determine whether math anxiety was related to working memory (WM) updating performance and, specifically, to the retrieval and substitution components of updating. A set of WM updating (WMU) tasks that involve different retrieval and substitution requirements were administered to 114 university students. In addition, participants completed a math anxiety assessment on two occasions: 1–2 weeks before and immediately prior to task administration to increase the likelihood of observing the relationship between math anxiety and updating performance. The results showed a relationship between math anxiety scores and updating performance. Math anxious individuals took longer and made more errors, especially on tasks that required retrieving information from WM. These results suggest that math anxious individuals are less efficient when it comes to accessing numerical information in WM. Consequently, they may struggle with math-related tasks that involve retrieving numerical information from WM.

## Introduction

Math anxiety is a negative emotional feeling associated with the manipulation of numbers and mathematical problem solving. This type of anxiety has a detrimental impact on scores in mathematical tests, leads to math avoidance and determines choices made at school (Hembree, 1990; Ashcraft and Ridley, 2005; Dowker et al., 2016). Numerous studies employing numerical tasks have shown specific difficulties of math-anxious individuals that may have their origin in representational, attentional-control or working memory (WM) deficits (Suárez-Pellicioni et al., 2016).

One consequence of math anxiety is its debilitating effect on cognitive performance and, specifically, on WM functioning (see Passolunghi et al., 2019 for an overview). WM is a system responsible for maintaining representations available for cognitive processing and is involved in numerous complex and everyday tasks. WM plays a significant role in mathematical performance (see Peng et al., 2016 for a review). For instance, when students undertake mathematical operations or solve problems, they must maintain some information in WM while perform the necessary steps to reach the solution. Ashcraft and Kirk (2001) conducted a seminal study that demonstrated how high math-anxious individuals exhibited lower performance on numerical WM tasks in comparison to peers with lower math anxiety levels. In the first experiment, participants were administered a computation span task; they were shown sets of arithmetical problems (e.g. 5 + 2 = ?, 9 − 6 = ?) and they had to solve the operations, give the answers (7, 3) and then recall the last number for each problem (2, 6). They were also administered a listening span task that entailed similar processes but included linguistic instead of numerical content. The authors found that math anxiety was specifically related to computation span and not to listening span performance. Thus, math anxiety may reduce effective WM capacity and hence mathematical performance.

Since then a number of studies have confirmed impaired WM performance related to math anxiety (e.g. Miller and Bichsel, 2004; Passolunghi et al., 2016; Shi and Liu, 2016; Trezise and Reeve, 2016; Justicia-Galiano et al., 2017). Indeed, some authors have argued that WM could mediate the relationship between math anxiety and math achievement (Hartwright et al., 2018). Math anxious individuals would be prone to experience difficulties in tasks that involve storing and retrieving information in WM, performing mental operations and updating intermediate results. This pattern is similar to that identified in the more studied relationship between WM and general anxiety. Moran (2016) conducted a review and meta-analysis of 177 samples which evidenced that anxiety was reliably associated with lower scores on WM tasks, yielding a small-to-moderate effect size (see also Visu-Petra et al., 2012 for a review).

Theories that have attempted to explain the association between anxiety and cognitive performance share the idea that anxiety-related processes interfere with cognitive resources. One influential approach to anxiety and cognition is the Attentional Control Theory (ACT; Eysenck et al., 2007; Eysenck and Derakshan, 2011). ACT states that anxiety reduces attentional control allocating resources to threat-related stimuli and decreasing the attentional focus on the current task (Eysenck et al., 2007). As a consequence of this competition, that presumably occurs at the level of executive functioning, it became more difficult to access to the severely limited WM resources. Thus, worrisome thoughts in high anxious individuals induce a functional reduction in WM capacity. More specifically, this proposal assumes that anxiety is associated with deficits in executive functioning as outlined by Miyake et al. (2000), and particularly with the shifting and inhibition functions. According to the ACT, anxiety may affect updating function under high cognitive load or in threatening situations. We are not aware, however, of any research that has explored the possible relationship between updating performance and math anxiety. Although math anxiety has been found to be related to WM performance that, in turn, is highly related to updating performance (Miyake et al., 2000), an appropriate evaluation of the possible relationship between updating and anxiety should be based on specific updating tasks.

Updating entails modifying content stored in WM to accommodate new information. This mechanism is critical for WM functioning which, due to its limited capacity, requires no longer relevant information to be continuously substituted. Indeed, updating is the executive function most closely related to WM (Miyake et al., 2000; Schmiedek et al., 2009), and is recruited by numerous complex cognitive tasks that involve a constant flow of incoming information. It is not surprising, therefore, that WM updating (WMU) is related to performance on arithmetical (e.g. Iuculano et al., 2011) and problem-solving tasks (e.g. Cornoldi et al., 2012).

The updating function is multifaceted. Ecker et al. (2010) identified three sub-processes or components found in various WMU tasks used in literature: retrieval, transformation and substitution. To illustrate these components, let us take the following scenario: a customer goes to the checkout with two items, having mentally calculated the total. They then pick up a third item. To keep track of how much they will have to end up paying, the customer has to retrieve the previous amount, add the price of the last product and substitute the previous partial sum for the updated amount. The first WMU component, retrieval, means accessing the to-be-updated information. In the example above, the individual has to retrieve the previous sum from WM in order to add the cost of the new item. Transformation refers to any process that changes information held in WM (i.e. the arithmetical operations in the example). The last component, substitution, means replacing the WM content with new information. In the example given, the customer has to store the result of the sum for future use (i.e. to pay). In a narrow sense, WMU would be identified with the substitution process that involves the replacement of the information. Indeed, substitution is a key component common to all the updating tasks while retrieval may be absent in some of them (Ecker et al., 2010). The focus of the current study was on the retrieval and substitution components.

Despite the relevance of the updating function for WM and the relationship between math anxiety and WM performance, to our knowledge, there are no studies that address whether or not math anxiety is related to performance on WMU tasks. Therefore, it is deemed relevant to specifically determine the possible relationship between WMU and math anxiety. If such relationship exists, it would be valuable to determine whether, and to what extent, performance on the different WMU components is related to math anxiety. Further knowledge about this relationship would contribute to a better understanding of individual differences in math learning.

In the present study, we sought to examine whether math anxiety is related to WMU and, specifically, to two of the aforementioned WMU components involved in updating information: retrieval and substitution. To achieve this, we developed different versions of a WMU task previously employed by Salthouse et al. (1991) and Oberauer et al. (2003). A highly convenient feature of this task for the present study is that it employs numerical information and operations with which math-anxious individuals encounter difficulties (Suárez-Pellicioni et al., 2016). Indeed, Namkung et al. (2019) have suggested that the relationship between math anxiety and WM is domain-specific: only the tasks that involve numbers would induce math anxiety that in turn disrupts the functioning of WM.

Four numerical tasks were designed which would include different combinations of the substitution and retrieval components (see Ecker et al., 2010; Linares et al., 2016, for a similar approach). Each task was assigned the initial(s) of the corresponding component(s): R for retrieval, lowercase t for transformation and S for substitution. It should be noted that this study focused solely on the retrieval and substitution components, meaning that the transformation component was not manipulated (this is indicated by lowercase t). Indeed, arithmetical operations were present in all conditions as a way of obtaining new values that could be either substituted or retrieved. The task that incorporated all components (RtS) required participants to retrieve numbers associated with a box on the screen (e.g. 5); apply a simple arithmetical operation to these numbers (e.g. + 1); and substitute the previous number linked to the box with the result of the operation (e.g. 6). The other tasks resulted from omitting components. The second subtask (Rt) involved retrieving a previously memorised number and applying an operation. The third subtask (tS) involved performing an arithmetical operation (e.g. 2 + 1) and replacing a previous value with the result. The t task meant simply carrying out arithmetical operations without retrieval or substitution requirements.

We assessed math anxiety in two different situations to determine its possible relationship with WMU and its components. The first assessment took place 1–2 weeks prior to experimental task administration in a neutral setting. The second assessment was carried out immediately before task execution, once participants were given the instructions and knew that they would have to do arithmetical operations and memorise numbers. Therefore, the experimental setting could be a potentially threatening situation for math-anxious individuals. This second assessment of math anxiety, in addition to providing a concurrent measure, could increase the possibility of observing the expected association between math anxiety and WMU performance.

In light of the above reviewed studies showing that math anxiety predicts poorer performance on measures of WM, and on the premise that updating is the executive function most closely related to WM, we would expect to observe WMU performance decrements related to math anxiety. Regarding the specific relationship between math anxiety and the retrieval and substitution components of WMU, previous studies have found that people with low WM capacity (Unsworth and Engle, 2008; Ecker et al., 2010) and younger children (Linares et al., 2016) exhibit certain difficulties when it comes to retrieving information from WM. Therefore, it could be hypothesised that math anxious individuals, who are prone to show low WM performance, could be less efficient at updating information in WM and specifically when information has to be retrieved from WM. We have not, however, made specific predictions about the possible link between math anxiety and substitution, given that some studies have reported no significant relationship (Ecker et al., 2010; Linares et al., 2016), while others have found an association between a sub-component of substitution and WM capacity (Singh et al., 2018).

## Materials and Methods

### Participants

A total of 114 psychology students (20 males and 94 females), aged 18–35 years (M = 19.85, SD = 2.54) volunteered in the study. They gave written informed consent prior to the first session and received course credit for their participation.

It was not possible to perform *a priori* power analysis, as there were no antecedents using the updating tasks with math anxious individuals. The sample size of about 110 participants was informed by previous studies focused on the relationship between anxiety and WM or updating related mechanisms (Gustavson and Miyake, 2016; Mammarella et al., 2018). This study was approved by the Research Ethics Committee of the University of Jaén and it was performed in accordance with the Declaration of Helsinki.

### Materials and Procedure

Participants attended two sessions between 1 and 2 weeks apart. In the first session, they were administered the Math Anxiety Rating Scale-Revised (MARS-R; Hopko, 2003) during regular classroom time. In the second session, groups of between 10 and 20 students completed the Single-Item Math Anxiety (SIMA) Scale (Núñez-Peña et al., 2014) and performed four cognitive tasks designed to assess the different WMU components. Participants were asked to respond to the SIMA after receiving the initial instructions for the WMU tasks and immediately before performing them. We choose the SIMA for the second MA assessment as a valid and efficient way to assess MA in the short interval between the instructions and the administration of the tasks.

#### Math Anxiety Questionnaires

The MARS-R is a 24-item instrument that assesses anxiety in math-related academic situations (e.g. watching a teacher work an algebraic equation on the blackboard). Responses are given on a five-point Likert scale ranging from 1 (no anxiety) to 5 (high anxiety). Scores on the MARS-R range from 24 to 120. The instrument showed high internal consistency as reflected by the Cronbach’s alpha for the original instrument (α = 0.98) and for the current study (α = 0.94).

The SIMA was employed as a measure of math anxiety in a math assessment context. It covers one item: “On a scale from 1 to 10, how math anxious are you?,” which is answered on a 10-point Likert scale from 1 (not anxious) to 10 (very anxious). Reliability estimates reported in the original study using different methods ranged from 0.63 to 0.78, and test–retest reliability was *r* = 0.81.

#### Working Memory Updating Tasks

Four tasks emerged from the orthogonal manipulation of the retrieval and substitution components. The factorial design’s underlying logic was to obtain estimates of the retrieval and substitution effects deriving from combinations of tasks that involved or did not involve each component.

**FIGURE 1:**
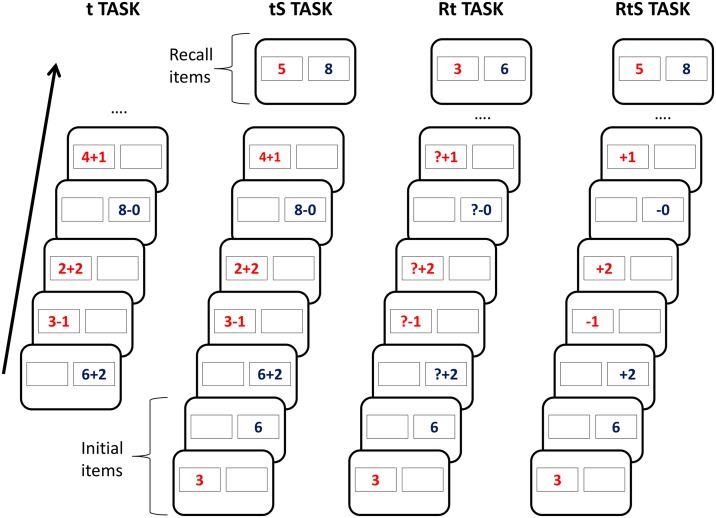
Example list for each task included in the present study. The “t” task required participants to perform arithmetical operations that appeared in red or blue inside two boxes. The “tS” task involved performing arithmetical operations and memorising the result for each box. Participants had to recall the last result for each box at the end of the list. The “Rt” task consisted of memorising two initial numbers for their corresponding box. Then, for each item, the participant had to retrieve the value for the box and use it as the first operand for the operation presented. The initial numbers were to be recalled at the end of the list. The “RtS” task involved memorising two initial numbers for each box. Following that, different arithmetical operations had to be applied to the number memorised for the corresponding box. The result for each operation had to be memorised for future use and, at the end of the list, the last two results for each box were to be recalled. See the text for further details.

A series of arithmetical operations appeared consecutively in two boxes across all tasks. Depending on the task, participants were or were not required to retrieve information associated with the box, apply the operation and then substitute, or not, the result for the corresponding box. It should be noted that all tasks involved operations since transformation was not manipulated in this study. Figure 1 shows an example list for each task.

The t task did not require retrieval or substitution. Simple operations (e.g. 3 − 1) were presented successively in their respective boxes and participants had to calculate and type the result without having to retrieve or replace any information. It should be noted that initial items were not presented in this condition because there was no information to memorise.

In the tS task, which involved Substitution, two initial items associated with two boxes had to be memorised. Then, complete operations (e.g. 3 − 1) associated with one of the two boxes were presented. Participants had to perform the operation, memorise and type the resulting number for the corresponding box. At the end of the list, participants were asked to type the values for each box.

In the Rt task, which required retrieval, participants initially memorised two numbers associated with two boxes. Subsequently, different incomplete operations (e.g. ? − 1) appeared one at a time in one of the boxes. Participants were asked to retrieve the number memorised for that box, apply the operation and enter the result. At the end of the list, participants had to type the number associated with each box.

Finally, in the RtS task, which involved Retrieval and Substitution, incomplete operations (e.g. − 1) were presented after the initial values for each box. Participants were asked to retrieve the value associated with the box, perform the operation, type the result and finally replace the value of the box with the result obtained. At the end of the list, participants had to enter the values for each box.

The tasks comprised a total of 80 lists (20 per each WMU task) made up of two initial items (except lists in the t task) and a variable number of study items: from 4 to 10. Lists differed in length to prevent participants from anticipating the end of each list. Initial items (numbers between 2 and 9) had different colours (red first and blue second) and appeared inside one of two rectangular boxes (2.8 cm high and 4.2 cm wide) positioned 1.6 cm apart on the screen. There were two types of study items depending on the task. Study items for the t and tS tasks were complete operations featuring a digit number and an arithmetical operation (e.g. 3 − 1). The first operand was a number between 2 and 9, and the arithmetical operations were ± 0, ± 1, or ±2. Study items for the Rt and RtS tasks were incomplete operations in which the first operand was omitted. They could comprise a question mark and an arithmetical operation for the Rt task (e.g. ? − 1) or an operation only for the RtS task (e.g. − 1). As with the initial items, study items were presented at random in one of two boxes and could be red or blue (see Figure 1).

For each item, participants had to type the number (in case of initial numbers) or the result of applying the operation (in case of study items). Response times and errors for each study item were recorded. At the end of the list, participants had to type the number memorised to each box. Feedback about the number of items answered correctly was provided at the end of each list.

A computer programme using E-prime 2.0 (Schneider et al., 2002) selected the initial items at random, determined the arithmetical operation for the study items and specified the box in which the item would be presented. The programme also calculated the value for each item that depended on the previous value for the corresponding box and the arithmetical operation. A different set of lists were generated for each participant. Tasks were performed on laptops with a standard 15-inch screen.

Lists for different tasks were administered preceded by two additional practice lists. There were two blocks that comprised 10 lists for each task. The first block included the subtasks in the following order: t, tS, Rt and RtS, whereas in the second block, the order was reversed: RtS, Rt, tS and t.

#### Analysis

Data were analysed using linear mixed-effects models with the lme4 package (Bates et al., 2014) in the R environment (R Core Team, 2016). This approach makes it possible to consider the entire sample and avoids the dichotomisation of the anxiety scores. In addition, these models have the advantage of including a random-effect structure that accounts for participants’ variability. We started by determining the random-effects structure that included a random intercept for participants and random slopes for retrieval and substitution. After, the fixed effects were assessed. All predictors (retrieval, substitution and math anxiety) and all two- and three-way interactions were entered in an initial model. Following a step-wise procedure, reduced models were specified by removing non-significant predictors. For each step, a reduced model was tested against the previous model using a log-likelihood ratio test. This analysis strategy was applied iteratively until the simplest model with adequate explanatory power was achieved. We conducted two sets of analyses, one for response times and one for errors, to test whether math anxiety interacted with the retrieval and substitution effects.

## Results

Only response times and errors for study items were included in the analysis. Response times from practice lists and from the incorrectly recalled lists (13.76%) were discarded from analyses. Response times lower than 200 ms and higher than the participant’s mean by more than 3.5 standard deviations were also excluded. This represented 1.01% of all observations from correct lists. Both anxiety measures were standardised to z-scores prior to analysis. Table 1 shows means, standard deviations and correlations among the variables considered in this study.

**TABLE 1:** Descriptive statistics and zero-order correlations between math anxiety measures, response times and errors on each task.

	**Mean**	***SD***	**MARS-R**	**SIMA**	**Time (t)**	**Time (tS)**	**Time (Rt)**	**Time (RtS)**	**Errors (t)**	**Errors (tS)**	**Errors (Rt)**	**Errors (RtS)**
MARS-R	69.17	17.16	–									
SIMA	6.09	2.19	0.68**	–								
Time (t)	1286.40	236.30	0.25*	0.31**	–							
Time (tS)	1639.61	360.71	0.25*	0.31**	0.78**	–						
Time (Rt)	1793.10	390.13	0.29*	0.39**	0.65**	0.75**	–					
Time (RtS)	1739.93	391.95	0.33**	0.43**	0.58**	0.79**	0.76**	–				
Errors (t)	2.66	3.57	–0.18	–0.05	0.08	0.08	0.12	0.08	–			
Errors (tS)	1.97	3.47	–0.13	–0.08	0.06	0.04	0.04	0.03	0.84**	–		
Errors (Rt)	8.48	8.20	0.15	0.23*	0.16	0.13	0.27*	0.26*	0.12	0.12	–	
Errors (RtS)	13.37	14.92	0.12	0.13	0.14	0.09	0.16	0.32**	0.16	0.24*	0.33**	–

* *p* < 0.05, ** *p* < 0.001.

First, we determined whether math anxiety was related to response times and interacted with the retrieval and substitution effects. Table 2 shows the parameter estimates for the final models with the MARS-R and the SIMA Scale. Model fit statistics are provided in Supplementary Table S1, and the results of model comparisons are presented in Supplementary Table S2.

**TABLE 2:** Estimates of fixed effects on response times and errors for each measure of math anxiety.

**Dependent variable**	**Anxiety measure**	**Effect**	**Estimate (*SE*)**	***df***	***t***	***p***	**95% *CI***
Time	MARS-R	Intercept	1286.40 (25.27)	135.38	50.90	<0.001	[1236.87, 1335.93]
		Retrieval	506.71 (25.02)	207.53	20.25	<0.001	[457.67, 555.75]
		Substitution	355.62 (23.51)	215.81	15.13	<0.001	[309.54, 401.70]
		MA	63.92 (24.16)	114.19	2.65	0.009	[16.57, 111.27]
		Retr × Subst	−403.14(30.19)	113.67	–13.35	<0.001	[-462.31, -343.97]
		Retr × MA	42.76 (19.93)	113.72	2.15	0.034	[3.70, 81.82]
	SIMA	Intercept	1286.42 (24.84)	136.21	51.79	<0.001	[1237.73, 1335.11]
		Retrieval	506.72 (24.59)	210.61	20.61	<0.001	[458.52, 554.92]
		Substitution	355.59 (23.51)	216.62	15.13	<0.001	[309.51, 401.67]
		MA	82.10 (23.65)	114.05	3.47	<0.001	[35.75, 128.45]
		Retr × Subst	−403.08(30.19)	114.96	–13.35	<0.001	[-462.25, -343.91]
		Retr × MA	63.44 (19.39)	112.91	3.27	0.001	[25.44, 101.44]
Errors	MARS-R	Intercept	2.66 (0.57)	296.30	4.65	<0.001	[1.54, 3.79]
		Retrieval	5.81 (1.06)	207.10	5.46	<0.001	[3.73, 7.90]
		Substitution	−0.69(0.89)	261.30	–0.78	0.438	[-2.45, 1.06]
		MA	−0.57(0.40)	321.80	–1.44	0.151	[-1.35, 0.21]
		Retr × Subst	5.59 (1.11)	341.90	5.03	<0.001	[3.41, 7.77]
		Retr × MA	1.83 (0.80)	155.90	2.30	0.023	[0.27, 3.39]
	SIMA	Intercept	2.66 (0.57)	292.30	4.64	<0.001	[1.54, 3.79]
		Retrieval	5.81 (1.06)	207.50	5.48	<0.001	[3.73, 7.89]
		Substitution	−0.69(0.90)	260.00	–0.77	0.440	[-2.45, 1.06]
		MA	−0.22(0.40)	324.00	–0.56	0.576	[-1.00, 0.56]
		Retr × Subst	5.59 (1.11)	342.00	5.03	<0.001	[3.41, 7.77]
		Retr × MA	2.11 (0.79)	157.50	2.67	0.008	[0.56, 3.66]

The models that included MARS scores as a math anxiety trait index revealed that response times increased when information had to be retrieved or substituted as denoted by the estimates of each effect (507 and 355 ms). The interaction between both WMU components indicated that the substitution effect was larger when the information had to not be retrieved. The negative estimate of the interaction reveals that when retrieval was required, response times were shorter in the RtS task, that involved substitution, than in the Rt task in which the information did not have to be replaced. This result replicates the finding reported in a previous study with other version of the tasks (Linares et al., 2016). Our explanation is that in the Rt task, participants have to refresh or rehearse the initial number after performing each operation and typing the result, given that the initial number has to be reused for future operations. In contrast, in the RtS task, as soon the operation is performed, the new result can be stored. The rehearsal step is not necessary for this task because the previous number may be discarded.

**FIGURE 2:**
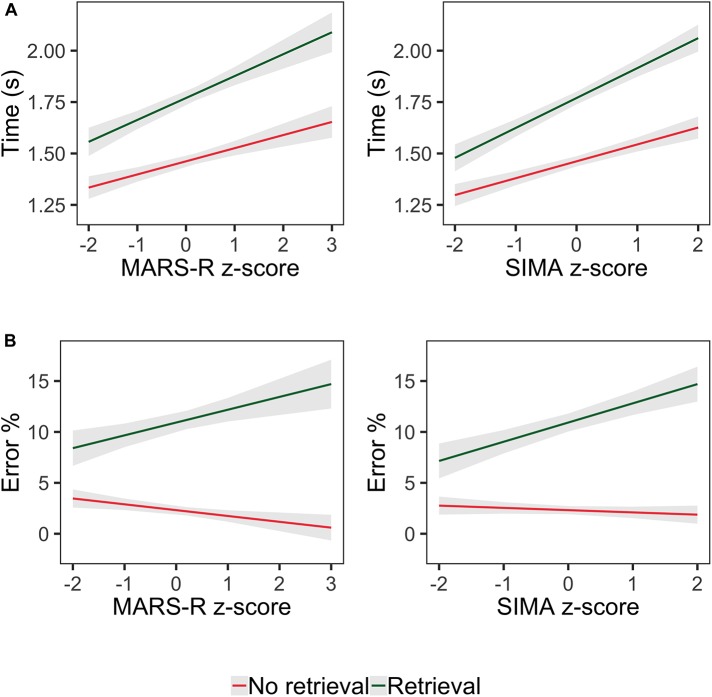
Estimated response times **(A)** and estimated errors **(B)** as a function of retrieval and math anxiety z-scores on MARS-R and SIMA. Shaded areas demarcate two standard errors.

More importantly, response times increased with anxiety assessed by MARS-R and there was a reliable interaction between retrieval and the anxiety score. This interaction was predicted by our hypothesis that anxious individuals would show difficulties when faced with the retrieval WMU component. Indeed, the interaction revealed that the retrieval effect became larger as the anxiety score increased. Specifically, retrieval time increased by 43 ms as math anxiety increased by a standardised unit (see Figure 2A).

The analogous analysis for the model that included the SIMA score showed similar effects and interactions (see Table 2). It is worth noting that the interaction between the SIMA score and retrieval was also found. As can be seen in Figure 2A, this interaction was explained by a larger retrieval effect as anxiety increased. Retrieval time increased by 63 ms as math anxiety assessed by the SIMA increased by a standardised unit.

In a second set of analyses, we examined whether math anxiety and substitution and retrieval had an effect on errors. Table 2 presents the parameter estimates for the final models. Supplementary Table S1 provides model fit statistics and Supplementary Table S2 shows the results of model comparisons.

Retrieval had a similar effect to that observed for response times, given that more errors were observed when the information had to be retrieved. The significant interaction between both WMU components showed that substitution induced more errors but only in the retrieval condition. Regarding the relationship with math anxiety, similar to the interaction previously described for response times, the retrieval effect increased as participants’ MARS scores increased (see Figure 2B). Specifically, retrieval errors increased by 1.8 as math anxiety increased by a standardised unit. The parallel analysis conducted using the SIMA scores revealed a very similar pattern of results. Again, an important result was the interaction between this anxiety measure and retrieval. Figure 2B shows that the retrieval effect was more pronounced as math anxiety increased.

As there were two blocks in which all tasks were presented in different orders, we tested a possible block effect by including it as a predictor in the models. The block effect was found to be significant for response times corresponding to the model with either MARS (β = −153.88, *SE* = 16.11, *p* < 0.001) or SIMA (β = −153.87, *SE* = 16.10, *p* < 0.001). Negative coefficients for the block effect indicate a practice effect. More importantly, no interactions between block and the remaining predictors held in the final models for response times. As for errors, neither the block effect nor the interactions between block and the other variables held in the final models.

## Discussion

This study aimed to investigate the relationship that math anxiety has with WMU and, specifically, with the retrieval and substitution components involved in WMU tasks. Results showed that math anxiety scores predict poorer WMU performance. Regarding the WMU components, math anxiety was negatively related to retrieval but not to substitution.

This study builds on the existing literature concerning the relationship between math anxiety and WM performance (e.g. Ashcraft and Kirk, 2001), providing clear evidence that math anxiety is also negatively related to performance in WMU tasks. From an ACT perspective, the debilitating effects of anxiety on updating should be weaker than with other executive functions (i.e. inhibition or switching) unless updating is evaluated in threatening situations (Eysenck et al., 2007). It should be noted that this view was based on earlier findings from span tasks, whereas the tasks included in the present study engaged specific updating components. Therefore, the current tasks may have proven more successful at revealing the effect of anxiety on updating performance.

Math anxiety affected response times. This result is in line with the predictions by the ACT theory that assumes that anxiety would impair performance efficiency (i.e. time) more so than performance effectiveness (i.e. accuracy). As proposed by ACT, longer times indicate that anxious people allocate extra processing resources to the task in order to maintain the level of accuracy.

The current study observes a similar relationship pattern between performance measures and math anxiety at both times of assessment: 2 weeks prior to experimental task administration in a neutral setting and immediately before task execution. It could be argued that the second situation could be perceived as more stressful and threatening than the first one because participants knew that they would shortly have to perform demanding tasks with numerical operations. It may be also that the assessment would lead to an increase in anxiety by making participants more aware of their actual level of anxiety. The results for both measures were, however, similar. Given the analogous pattern observed for both measures, we have no evidence to conclude that different mechanisms are involved in the relationship between both appraisals of anxiety and WMU.

An additional key novel finding of this study is that math anxiety was related to WMU performance, especially when information had to be retrieved. As math anxiety increased, so did the time spent retrieving information from WM. If anxiety induced a general reduction in processing speed, its effect would have been analogous in both retrieval conditions. On the contrary, the differential impact of anxiety on both conditions indicates that this effect is not merely due to a general slowing down in information processing, but is specifically related to retrieval from WM. This slowing down to retrieve information in WM may be a shared characteristic among people who suffer from other types of anxiety. Visu-Petra et al. (2009) studied response timing patterns in children with trait anxiety informed by their parents. They found that trait anxiety was related to the length of interword pauses at recall that would reflect search and retrieval operations in WM.

Further support for the idea that math anxiety impairs retrieval in updating tasks comes from the analysis of errors. Errors increased with anxiety when retrieval was required, but they remained low when retrieval was not necessary. It should be noted that retrieval speed from WM may ultimately affect recall accuracy. Indeed, the errors were moderately correlated with response times in the retrieval conditions. A slower retrieval process would increase the likelihood of information degrading over time as a result of interference. Together, the results suggest that anxious people are less likely to have representations held in WM available, or that they are less effective during retrieval.

Working memory retrieval difficulties related to math anxiety may ultimately come down to a number of reasons. First, retrieval difficulties could be indicative of the poor quality of numerical representations held in WM by math anxious individuals. This problem could be shared by children with math difficulties who also demonstrate lower performance in updating tasks involving numerical information (Iuculano et al., 2011; Pelegrina et al., 2015). Second, retrieval problems may be due to a weaker binding between context cues (boxes and colours) and content (numbers). Third, the retrieval difficulties found could also point to an increased susceptibility to interference among content held in WM. This interpretation is consistent with other recent studies demonstrating that high trait anxiety individuals have difficulties dealing with interference from distractors coming either from content in WM or from visually presented information (Hoshino and Tanno, 2016).

With respect to the substitution component of updating, although this process had a clear effect on response times, it was not related to math anxiety. The absence of a relationship between substitution and math anxiety is in line with results from recent studies that did not observe individual differences in WM or age-related differences associated with substitution performance (Ecker et al., 2010; Linares et al., 2016). Nonetheless, Singh et al. (2018) have reported that the efficiency of a sub-component of substitution, namely the removal of no-longer relevant information, relates to WM capacity. There is some evidence that MA may interfere with inhibitory processes. Gustavson and Miyake (2016) found that trait worry is associated with longer response times to irrelevant information that should have been inhibited. In a similar vein, math-anxious children show lower performance on a proactive interference task (Mammarella et al., 2018) and make more intrusion errors in a verbal WM task (Passolunghi et al., 2016). Given the low rate of errors shown for the task that required only substitution in the present study, it could be the case that this task made lower executive demands than the tasks which involved retrieval. Future research might use tasks that place greater demands on the substitution component in order to better clarify its possible relationship to math anxiety.

There are some considerations regarding the two WMU components studied. First, on the basis of the task analysis conducted by Ecker et al. (2010), substitution is the unique component process common to all the updating tasks. Given that no relationship between substitution performance and MA was observed, it could be argued that MA does not affect updating. It should be taken into account, however, that MA was related to retrieval. This component is involved in the majority of updating tasks and makes an independent contribution to WMU performance. Therefore, it can be concluded that MA influence WMU by interfering with retrieval. Second, the WMU components described in this report are not exclusive to updating tasks, as these processes may be engaged, for instance, in WM or in some complex numerical tasks. This implies that difficulties in retrieving information may in part explain the poorer performance on WM tasks by math anxious individuals observed in previous studies (e.g. Miller and Bichsel, 2004; Passolunghi et al., 2016; Justicia-Galiano et al., 2017). Given that retrieval may be engaged both in WMU and in WM tasks, further empirical work is required to determine the shared and unique associations of both WM and WMU with math anxiety.

There are some limitations to this study that could be addressed in future research. The fact that we have only used a series of tasks based on numerical information is particularly relevant. Numerical stimuli may be associated with negative and threatening valence for math-anxious individuals (Rubinsten et al., 2015); thus, the possible deleterious effects of math anxiety could be greater for tasks featuring threat-related stimuli. Some studies have shown that math anxiety difficulties are specifically related to tasks with numerical information (Ashcraft and Kirk, 2001) or to dysfunctional math-related contexts (Shi and Liu, 2016). In fact, Namkung et al. (2019) in a recent meta-analysis pointed out that only those WM tasks that involve numerical information may prompt math anxiety. As such, the results in the present study may not be generalised to other types of information. It would, therefore, be desirable to examine the link between WMU and anxiety using different types of materials.

Future research should also control for general anxiety (see e.g. Rubinsten and Tannock, 2010; Mammarella et al., 2015). WM performance has been related positively, albeit moderately, to general anxiety. Although math and general anxiety are considered different constructs (Hembree, 1990), it would be important to determine the extent to which the retrieval difficulties in WMU tasks are related to each type of anxiety.

Finally, we cannot make causal claims based on the present study. Despite having adopted the prevalent view about the direction of the relationship between math anxiety and WM, that is, math anxiety undermines WM availability and updating effectiveness, the opposite direction is also a possibility (see Moran, 2016), and it may also be the case that WM and anxiety influence each other iteratively over a period of time (Trezise and Reeve, 2016). It would be interesting to include math-anxiety manipulations in order to determine whether they are capable of affecting WMU performance.

## Conclusion

The current study has provided evidence that math anxiety is negatively related to performance in numerical WMU tasks and, in particular, to difficulties in retrieving numerical information that has to be updated. Therefore, math anxious individuals would likely struggle with math-related tasks depending on the updating requirements of these tasks. The information provided by the present study might be relevant to educational settings, because updating is required for numerous school-related tasks such as mental calculation or problem-solving. Therefore, our study could contribute to the design of interventions aimed at ameliorating some cognitive difficulties of math-anxious individuals by considering the retrieval demands of tasks that require updating. For instance, an approach could involve reducing the burden due to updating on math exercises and tasks. Training these processes could be another possibility.

## Data Availability Statement

The datasets used and analysed during the current study are available in the OSF repository [osf.io/w5qx4].

## Ethics Statement

The studies involving human participants were reviewed and approved by Comité de Ética de la Universidad de Jaén. The participants provided their written informed consent to participate in this study.

## Author Contributions

SP conceived and designed research, performed most of the data analysis and wrote the main manuscript text. MJ-G and MM-P contributed to the experimental design and conducted the experiment. RL contributed to data analysis and interpretation and prepared figures for the manuscript. All authors read, reviewed and approved the manuscript.

## Conflict of Interest

The authors declare that the research was conducted in the absence of any commercial or financial relationships that could be construed as a potential conflict of interest.
