# Supplementary material for: Math Anxiety and Working Memory Updating: Difficulties in Retrieving Numerical Information From Working Memory
Source: Front Psychol. 2020 Apr 9;11:669. doi: 10.3389/fpsyg.2020.00669 (PMC7160366; doi:10.3389/fpsyg.2020.00669)
Supplement: Supplementary file 1 [file Table_1.DOCX]

Math anxiety and working memory updating: Difficulties in retrieving information from working memory

Santiago Pelegrina, M. José Justicia-Galiano, M. Eva Martín-Puga, Rocío Linares

Supplementary material

Supplementary Table 1. Model fit statistics for the linear-mixed-effect models predicting response times and errors for each measure of math anxiety

|  | | | SIMA | | | | |  | | MARS | | | | |  |
| --- | --- | --- | --- | --- | --- | --- | --- | --- | --- | --- | --- | --- | --- | --- | --- |
|  | Model | Fixed effects | | AIC | BIC | logLik | df | |  | | AIC | BIC | logLik | df | |
| Time | | | |  |  |  |  | |  | |  |  |  |  | |
|  | 0 | Retr + Subst + MA + Retr x Subst + Retr x MA + Subst x MA + Retr x Subst x MA | | 6228.6 | 6290.2 | -3099.3 | 15 | |  | | 6240.6 | 6302.2 | -3105.3 | 15 | |
|  | 1 | Retr + Subst + MA + Retr x Subst + Retr x MA + Subst x MA | | 6227.2 | 6284.7 | -3099.6 | 14 | |  | | 6238.9 | 6296.4 | -3105.4 | 14 | |
|  | 2 | Retr + Subst + MA + Retr x Subst + Retr x MA | | 6227.5 | 6280.9 | -3100.7 | 13 | |  | | 6238.6 | 6292.0 | -3106.3 | 13 | |
|  | 3 | Retr + Subst + MA + Retr x Subst + Subst x MA | | 6236.5 | 6290.0 | -3105.3 | 13 | |  | | 6242.2 | 6295.6 | -3108.1 | 13 | |
|  | 4 | Retr + Subst + MA + Retr x MA + Subst x MA | | 6348.6 | 6402.1 | -3161.3 | 13 | |  | | 6360.1 | 6413.5 | -3167 | 13 | |
|  | 5 | Retr + Subst + MA + Retr x Subst | | 6235.5 | 6284.8 | -3105.8 | 12 | |  | | 6241 | 6290.4 | -3108.5 | 12 | |
|  | 6 | Retr + Subst + MA + Retr x MA | | 6348.4 | 6397.7 | -3162.2 | 12 | |  | | 6359.4 | 6408.7 | -3167.7 | 12 | |
| Errors |  |  | |  |  |  |  | |  | |  |  |  |  | |
|  | 0 | Retr + Subst + MA + Retr x Subst + Retr x MA + Subst x MA + Retr x Subst x MA | | 3144.9 | 3206.7 | -1557.4 | 15 | |  | | 3146.1 | 3208.0 | -1558.1 | 15 | |
|  | 1 | Retr + Subst + MA + Retr x Subst + Retr x MA + Subst x MA | | 3142.9 | 3200.6 | -1557.4 | 14 | |  | | 3144.2 | 3201.9 | -1558.1 | 14 | |
|  | 2 | Retr + Subst + MA + Retr x Subst + Retr x MA | | 3140.9 | 3194.5 | -1557.4 | 13 | |  | | 3142.5 | 3196.1 | -1558.3 | 13 | |
|  | 3 | Retr + Subst + MA + Retr x Subst + Subst x MA | | 3146.2 | 3199.8 | -1560.1 | 13 | |  | | 3147.3 | 3200.9 | -1560.7 | 13 | |
|  | 4 | Retr + Subst + MA + Retr x MA + Subst x MA | | 3165.3 | 3218.9 | -1569.7 | 13 | |  | | 3173.8 | 3227.4 | -1573.9 | 13 | |
|  | 5 | Retr + Subst + MA + Retr x Subst | | 3145.8 | 3195.3 | -1560.9 | 12 | |  | | 3145.7 | 3195.2 | -1560.8 | 12 | |
|  | 6 | Retr + Subst + MA + Retr x MA | | 3163.3 | 3212.8 | -1569.7 | 12 | |  | | 3172.1 | 3221.6 | -1574.1 | 12 | |

*Note*. MA: Math anxiety; Retr: Retrieval; Subst: Substitution. The random effect was the same for all models: 1 + R + S | SUJ

Supplementary Table 2. Model comparisons of the linear-mixed-effect models predicting response times and errors for each measure of math anxiety

|  |  | MARS | | |  | SIMA | | |
| --- | --- | --- | --- | --- | --- | --- | --- | --- |
| Dependent  Variable | Comparison | *X^2^* | *df* | *p* |  | *X^2^* | *df* | *p* |
| Time | Model 0 vs. Model 1 | 0.31 | 1 | .581 |  | 0.62 | 1 | .430 |
|  | Model 1 vs. Model 2 | 1.72 | 1 | .190 |  | 2.28 | 1 | .131 |
|  | Model 1 vs. Model 3 | 5.29 | 1 | .021 |  | 11.35 | 1 | .001 |
|  | Model 1 vs. Model 4 | 123.19 | 1 | < 0.001 | | 123.46 | 1 | < 0.001 |
|  | Model 2 vs. Model 5 | 4.45 | 1 | .035 |  | 10.04 | 1 | .002 |
|  | Model 2 vs. Model 6 | 122.83 | 1 | < 0.001 | | 122.97 | 1 | < 0.001 |
|  | Model 0 vs. Model 2 | 2.02 | 2 | .364 |  | 2.91 | 2 | .234 |
| Errors |  |  |  |  |  |  |  |  |
|  | Model 0 vs. Model 1 | 0.10 | 1 | .756 |  | 0.03 | 1 | .864 |
|  | Model 1 vs. Model 2 | 0.30 | 1 | .587 |  | 0.00 | 1 | .994 |
|  | Model 1 vs. Model 3 | 5.10 | 1 | .024 |  | 5.33 | 1 | .021 |
|  | Model 1 vs. Model 4 | 31.61 | 1 | < 0.001 | | 24.44 | 1 | < 0.001 |
|  | Model 2 vs. Model 5 | 5.19 | 1 | .023 |  | 6.96 | 1 | .008 |
|  | Model 2 vs. Model 6 | 31.64 | 1 | < 0.001 | | 24.44 | 1 | < 0.001 |
|  | Model 0 vs. Model 2 | 0.39 | 2 | .392 |  | 0.03 | 2 | .986 |
